# Sepsis in elderly patients: the role of neutrophils in pathophysiology and therapy

**DOI:** 10.1007/s11739-023-03515-1

**Published:** 2024-01-31

**Authors:** Davide Ramoni, Amedeo Tirandi, Fabrizio Montecucco, Luca Liberale

**Affiliations:** 1 First Clinic of Internal Medicine, Department of Internal Medicine, University of Genoa, 6 Viale Benedetto XV, 16132 Genoa, Italy; 2 IRCCS Ospedale Policlinico San Martino Genoa—Italian Cardiovascular Network, Genoa, Italy

**Keywords:** Neutrophil, Sepsis, Elderly, Enflammaging

## Abstract

Sepsis is among the most important causes of mortality, particularly within the elderly population. Sepsis prevalence is on the rise due to different factors, including increasing average population age and the concomitant rise in the prevalence of frailty and chronic morbidities. Recent investigations have unveiled a "trimodal" trajectory for sepsis-related mortality, with the ultimate zenith occurring from 60 to 90 days until several years after the original insult. This prolonged temporal course ostensibly emanates from the sustained perturbation of immune responses, persevering beyond the phase of clinical convalescence. This phenomenon is particularly associated with the aging immune system, characterized by a broad dysregulation commonly known as "inflammaging." Inflammaging associates with a chronic low-grade activation of the innate immune system preventing an appropriate response to infective agents. Notably, during the initial phases of sepsis, neutrophils—essential in combating pathogens—may exhibit compromised activity. Paradoxically, an overly zealous neutrophilic reaction has been observed to underlie multi-organ dysfunction during the later stages of sepsis. Given this scenario, discovering treatments that can enhance neutrophil activity during the early phases of sepsis while curbing their overactivity in the later phases could prove beneficial in fighting pathogens and reducing the detrimental effects caused by an overactive immune system. This narrative review delves into the potential key role of neutrophils in the pathological process of sepsis, focusing on how the aging process impacts their functions, and highlighting possible targets for developing immune-modulatory therapies. Additionally, the review includes tables that outline the principal potential targets for immunomodulating agents.

## Introduction

Sepsis is currently defined as a life-threatening organ dysfunction caused by a dysregulated host response to infection, with organ dysfunction defined by Sequential Organ Failure Assessment (SOFA) score ≥ 2 [1]. This new definition marks a deep line from the traditional association of infection and inflammation, dismissing the old terms "systemic inflammatory response syndrome" and "severe sepsis". Septic shock represents a specific subset of sepsis, clinically characterized by the requirement of vasopressor therapy and serum lactate concentration exceeding 2 mmol/L. This condition is associated with high mortality rates, reaching up to 40% [1].

**Fig. 1:**
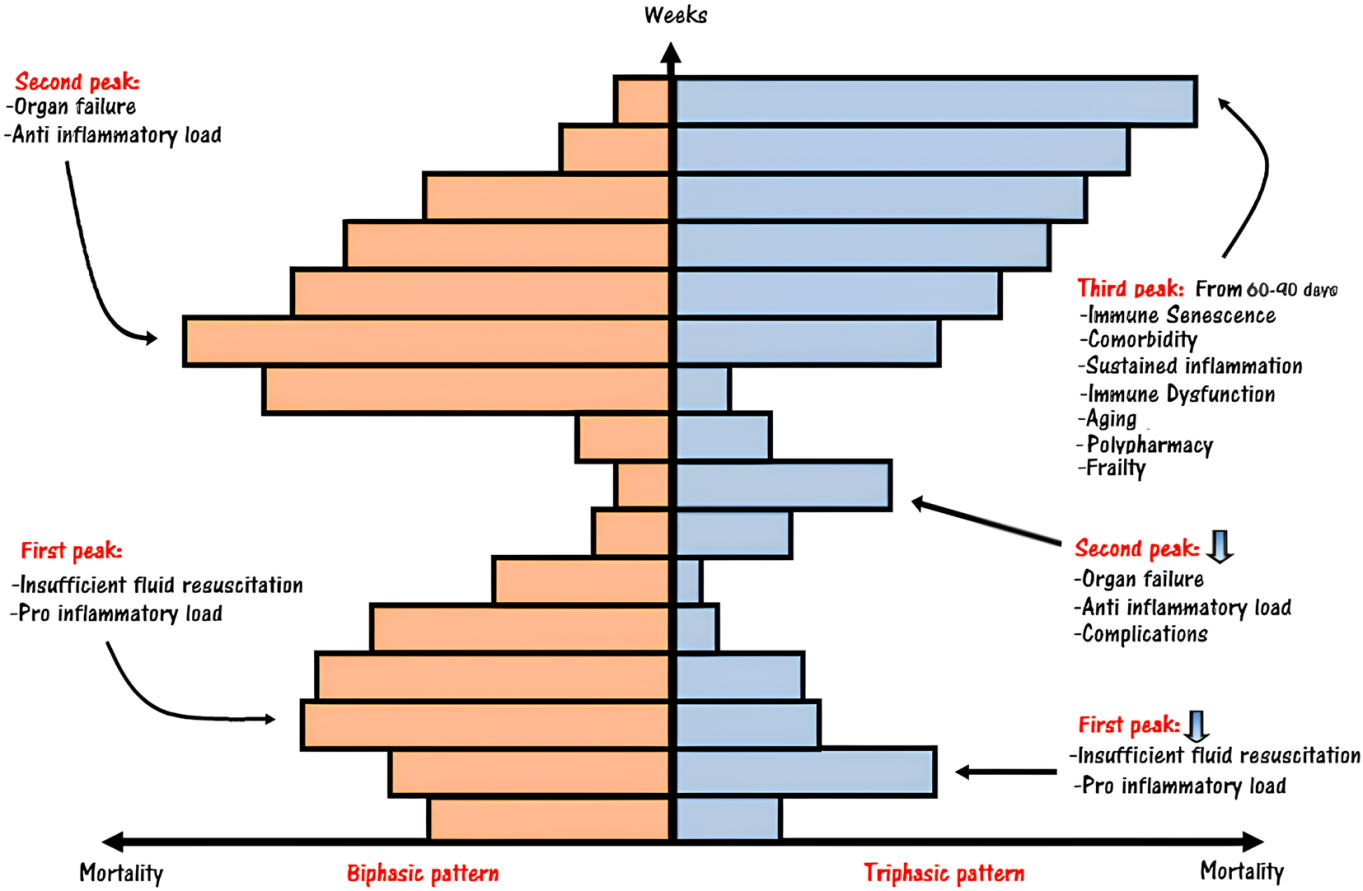
Sepsis mortality rates. Sepsis-related mortality was reported to have a biphasic pattern, characterized by an early first peak due to inadequate antibiotic therapy and fluid challenge and a second peak (from day 1 to first weeks) due to multi-organ failure. In the last years, thanks to the improvement of standard of care, especially in the intensive care unit department, the mortality rate showed a triphasic pattern. The third peak, starting from 60 to 90 days to several years, is more typical in a population of elderly subjects with the burden of multiple comorbidity

Previously, sepsis-related mortality was described as having a biphasic pattern, with an early peak attributed to inadequate fluid resuscitation and a late peak caused by persistent organ injury or failure. However, recent evidence has presented a shift toward a triphasic pattern for sepsis-related mortality [2]. Notably, advances in intensive care protocols, coupled with the increasing average lifespan, have likely reduced the impact of the early peak and brought attention to the emergence of a third peak. This third peak occurs from 60 to 90 days and can persist for several years after the onset of sepsis, as illustrated in Fig. 1.

The worldwide population of individuals aged 60 years or older is consistently increasing. In the late 1950s, this age group accounted for approximately 5% of the total population, but it is projected to rise significantly, reaching up to 21% by the year 2050 [3]. Elderly patients represent a significant portion of the deaths occurring during the third peak as they face a high risk of persistent, recurrent, secondary, or nosocomial infections. Among different pathophysiological changes occurring with age, a large number of predisposing factors (such as polytherapy and malnutrition), together with the surge of chronic low-grade systemic inflammatory response—the so-called “inflamm-aging”—deeply impact on the physiological response to microbial infections with the final effect of reducing survival rates [4–6].

The aging of the immune system, known as immuno-senescence, is considered a critical factor contributing to inflamm-aging. This process is believed to be responsible for the increased susceptibility of the elderly to infections and potentially to autoimmune diseases and cancer [7]. Although the precise pathophysiological mechanisms are not yet fully clarified, immunosenescence ultimately leads to dysregulation of immune functionality, favoring a chronic, low-grade, and sterile activation of innate immune cells. Consequently, the adaptive immune response becomes compromised, and the immune system struggles to respond effectively to appropriate immunogenic stimuli [8].

Given the critical role of innate immune cells—in particular, neutrophils—as the first line of defense against pathogens and their connection to the development of inflamm-aging, this review article aims to provide a comprehensive overview of the role of these cells in the pathophysiology of sepsis in the elderly population but also sheds light on potential targets for the development of immune-modulatory therapies.

## A shared common pathway between aging, chronic diseases, and sepsis

While the classic definition of the elderly population used to be set at 65 years old, more recent studies have shifted this age threshold to 75 years. The clinical management of elderly individuals during sepsis is more complex due to limited evidence (as most trials include younger subjects to avoid confounders), blurred symptoms, poly-therapy, and presence of comorbidities.

Aging, chronic diseases, and acute infections all share a common underlying mechanism: an abnormal and sustained inflammatory response that impairs the normal immune function. The elderly often experience a chronic low-grade inflammatory state, impacting their immune response and leading to constant hyper-stimulation. This persistent over-stimulation induces changes in immune cell behavior and differentiation, potentially explaining the progression and development of numerous chronic diseases and heightened susceptibility to infectious complications, particularly from opportunistic bacteria and fungi [9].

Also, this immune dysregulation contributes to decreased quality of life, increased hospital readmission rates, higher recurrence of infections, and poor survival outcomes [10]. On the other hand, sepsis can be regarded as an accelerator of immuno-senescence, the process of immune system aging, which promotes the development of chronic inflammatory diseases.

Apart from immuno-senescence, several other mechanisms have been identified as potential contributors to the onset of inflammaging. These mechanisms include increased production and reduced disposal of misfolded and misplaced proteins, epigenetic modifications, metabolic alterations, dysbiosis (imbalance in the gut microbiota), mitochondrial dysfunction, oxidative stress, and genetic predisposition. By highlighting these factors and their interconnectedness, we gain a deeper understanding of the complex dynamics between aging, inflammation, and sepsis in the elderly population. This knowledge can pave the way for better-targeted interventions and improved management strategies for sepsis in older adults.

A distinct time course of the immune system response is observed between young and elderly individuals [6]. While this phenomenon is often considered a para-physiological process, it can have detrimental effects in the elderly. During the initial pro-inflammatory response against an infection, if the pathogen is promptly eliminated, the immune system's homeostasis is quickly restored. However, in the case of sepsis or septic shock, especially in elderly patients, the dysfunctional immune system mounts a response that extends beyond pathogen eradication and can persist for months or even years, altering the immune system homeostasis. Therefore, the balance between immunosuppression and sustained inflammation may differentiate the course of infection, both at short and long term and is crucial to prevent the immune response from becoming counterproductive [5].

The similarities between immunosenescence in elderly patients and the immunosuppression induced by sepsis suggest common pathways that act synergistically. Nevertheless, a comprehensive theory that unifies these mechanisms is still lacking, and this gap contributes to the absence of specific targeted therapies for sepsis in older adults. In summary, understanding the differential immune responses in young and elderly individuals during sepsis is crucial for developing effective and tailored therapeutic strategies.

## How does immune function change in the elderly?

In younger adults, the innate immune system mounts a well-coordinated and controlled response to infections, effectively clearing the invading pathogens while minimizing host tissue damage. However, sepsis disrupts this immune response by altering the functionality and quality of effector cells [11].

As we age, the dysregulation of the innate immune system becomes more pronounced, leading to persistent inflammation and a range of impairments in immune cell function. This includes reduced cytokine production in response to appropriate antigenic stimuli, contracted antigen presentation, decreased phagocytosis and chemotaxis, altered reactive oxygen species (ROS) production, chronic catabolism, and defective cellular apoptosis. Among the many affected cell populations are polymorphonuclear neutrophils (PMN) (as summarized in Table 1). Despite the preservation of immune cell counts in the elderly even at extreme ages, these cells undergo age-dependent functional impairments [12].

**Table 1:** Comparison of major modifications of the innate immune system during sepsis or immuno-senescence

Innate immunity	Sepsis induced immuno-suppression	Immuno-senescence
*Pro-inflammatory cytokines*	Release of “cytokine storm” (↑ IL-1, TNF α, IL-6, Il-8, IFN)	Impaired cytokine production of IL-6, TNF α, IL-1
*Anti-inflammatory cytokines*	Increased IL-10, IL-7 production	Altered IL-10, IL-4 levels
*TLR*	Up-regulation of TLR expression TLR signal induced the “cytokine storm”	Dysregulated TLR signaling
*Apoptosis*	Impaired apoptosis (increased in macrophages and DCs, delayed in PMNs)	Decreased susceptibility to the damage-induced apoptosis
*Oxidative burst*	Increased oxidative burst	Altered oxidative burst
*Phagocytosis*	Reduced phagocytosis in macrophages, PMNs Complement activation of C5a	Defects in phagocytosis in macrophages, PMNs
*Migration*	Altered recruitment and chemotaxis	Altered recruitment and chemotaxis
*HLA*	Contracted antigen presentation in APC and decreased HLA-DR expression in monocytes	Decreased HLA-DR expression in monocytes
*NK cells*	Dysregulated activation of NK cells with exacerbates inflammatory bursting	Increased number of NK cells and decreased cytotoxic function
*DCs*	Impaired DCs functions (impaired cytokine production, reduced antigen expression, increased apoptosis)	Decreased stimulation of antigen-specific T cells, defective phagocytosis, decreased migration to lymph nodes
*Macrophages*	Impaired macrophages functions (altered ROS production, reduced phagocytosis, reduced antigen presentation, reduced chemotaxis)	Reduced MHC class II expression, reduced oxidative burst and reduced phagocytosis
*Endothelial cells*	Impaired endothelial cells (increased capillary permeability, promoted of pro-coagulation state, increased production of endothelial adhesion molecules)	Increased release of pro-inflammatory substances Impaired immuno-surveillance of senescent endothelial cells
*Neutrophils*	Impaired PMNs functions (impaired ROS production, reduced phagocytosis, reduced chemotaxis, impaired recruitment, altered NETs production) Decreased apoptosis (↑, Mcl-1, and BCL-xl) ↑ CCR2 driving PMNs inappropriate migration sites of infections Desensitization of CXCL12, ↓ CXCR4 in PMNs	Reduced antimicrobial activity, impaired oxidative burst Reduced neutrophil chemotaxis and migration Decreased phagocytosis Subset populations of PMNs increased “reverse” migration by ↑ CXCR4 Increased release of NETs
*Adaptive immunity*	Thymic involution Reduced CD4 T cell, altered adhesion molecules, increased apoptosis Reduced T_H_1 cytokine response Increased TH2 cytokine response Reduced number of B-cells and reduced antibody production	Thymic involution and decreased naive cells Reduced CD4:CD8 ratio Reduced T_H_1 cytokine response Increased TH2 cytokine response Reduced number of B-cells and reduced antibody production

*APC* antigen-presenting cell, *BCL-2* B-cell lymphoma 2 proteins, *BCL-xl* B-cell lymphoma extra large, *BIM* Bcl-2-like protein 11, *CCR2* C–C chemokine receptor type 2, *CD* cluster of differentiation, *CXCL-12* chemokine C-X-C motif ligand 12, *CXCR-4* C-X-C chemokine receptor type 4, *DC* dendritic cell, *HLA* human leukocyte antigen, *Mcl-1* myeloid cell leukemia 1, *MHC* major histocompatibility complex, *NK* natural killer, *IL* interleukin, *NET* neutrophil extracellular trap, *PMN* polymorphonuclear neutrophil, *ROS* reactive oxygen species, *T*_*H*_ CD4 + lymphocytes T Helper, *TLR* tool-like receptor, *TNF* tumor necrosis factor

Usually, in course of sepsis, the host's response to infection is initiated by the systemic activation of the innate immune system mainly via the interaction between pathogen-associated molecular patterns (PAMPs) and pattern recognition receptors (PRRs). Additionally, the release of endogenous damage-associated molecular patterns (DAMPs), traditionally associated with sterile inflammation, appears to have an emerging role in sepsis by perpetuating inflammatory signals and delaying damage resolution. Of interest, in the context of sepsis, DAMPs can be released by death cells but also actively by activated cells. PAMPs and DAMPs are then recognized by (pattern recognition receptors) PRRs resulting in numerous downstream effects designed to promote the host's immune response to the newly identified pathogen and resulting in a severe and persistent inflammatory response through the release of IL-1, TNF, IL-6, and IL-17 [14]. Although the cytokine storm is essential during the early phase of the host response against pathogens, these cytokines also hold the potential to cause extensive tissue damage when not properly regulated. Consequently, the cytokine storm is associated with the first peak of early mortality in sepsis [2].

In the elderly, the first phase of sepsis is typically altered both qualitatively and quantitatively. The release of pro-inflammatory cytokines lasts longer and is increased, favoring a higher degree of tissue damage [11, 14].

Subsequently, or even simultaneously, an anti-inflammatory compensatory state begins, involving immunomodulatory cytokines, such as IL-10, IL-4, and transforming growth factor beta-β [15]. In younger patients, this response results in reduced inflammatory and cellular activation, facilitating the resolution of the inflammatory response. However, in the elderly, this immunomodulatory response is defective and skewed toward chronic low levels of inflammation. Recent research suggests that the two phases likely overlap, and the strict distinction between pro- and anti-inflammatory cytokines may be too simplistic, as many cytokines exert opposite functions depending on the microenvironment [11].

As individuals age, innate immune cells show defective antigen-presenting functions, and macrophages exhibit reduced expression of Human Leukocyte Antigen—DR isotype (HLA-DR), leading to a blunted stimulation of major histocompatibility complex class II [16]. Further, the expression of toll-like receptors on various immune cells is decreased in the aged population, rendering them more susceptible to microbial pathogens. Furthermore, innate immune cells move their metabolism from oxidative phosphorylation toward an increase in aerobic glycolysis and consequent lactate production [17]. As a result, detecting high serum levels of lactic acid becomes a strong indicator of worse outcomes.

One major consequence of the hyper-inflammatory state is the generation of free radicals, produced by the activation of neutrophils, macrophages, and endothelial cells in response to pathogen contact or indirectly via cytokine-mediated activation. However, in the elderly, the production of ROS is impaired, resulting in an ineffective oxidative burst and reduced bactericidal capacity of PMN cells [18]. Concomitantly, this alteration disrupts the homeostatic balance and leads to lipid peroxidation and damage to DNA, proteins, and mitochondria [19].

Another impaired mechanism associated with aging is apoptosis, a crucial process in maintaining the balance between survival and death of damaged cells. Increasing evidence suggests that apoptosis is widely involved in immunosenescence. Accordingly, TNF-α and the typical sepsis-related cellular damage can enhance the apoptotic process, leading to alterations in T cell populations, increased risk of autoimmunity, and accumulation of effector/memory cells. Recent evidence indicates that the long-life, constant stimulation of the immune system and the oxidative burst can modify the behavior of apoptotic lymphocytes and antigen-presenting cells [20].

Lastly, in recent years, the role of inflammasomes has emerged as a critical signaling pathway in the innate immune system and the progression of inflammation. Inflammasomes are assembled upon PRR activation. Specifically, five protein receptors, including members of the nucleotide-binding oligomerization domain (NOD), leucine-rich repeat (LRR)-containing protein (NLR) family—particularly the pyrin domain-containing 1–3 and CARD domain-containing 4 members (NLRP1, NLRP3, and NLRC4)—as well as the proteins absent in melanoma 2 (AIM2) and pyrin, have been identified with the capability to assemble inflammasomes. PRR activation generates a complex that converts pro-inflammatory caspases into their mature form, inducing a significant release of pro-inflammatory cytokines. Notably, inflammasome activation of the active protease Caspase-1 triggers a downstream response, resulting in the release of IL-1β and IL-18. Additionally, activated Caspase-1 cleaves Gasdermin-D inducing pore formation on the membrane. Such mechanism facilitates cytokines release and can eventually lead to pyroptosis, a form of programmed cell death [21].

A study conducted in vitro and in vivo demonstrated that neutrophils, conventionally viewed as the target of IL-1β, can activate the NLRC4 inflammasome during acute Salmonella infection [22]. Here, neutrophils emerge as major producers of IL-1β in vivo, with distinctive resistance to undergo pyroptosis. This enables neutrophils to support IL-1β production at the infection site without compromising their essential antimicrobial function, which would be lost if they rapidly underwent cellular lysis after Caspase-1 activation. Accordingly, after infection, resident macrophages react to infection by releasing a swift burst of IL-1β within the initial hour, whereas neutrophils might maintain IL-1β levels in the subsequent hours [22].

A growing body of evidence indicate that in the elderly, the NLRP3 inflammasome is overactivated. Studies on elderly patients infected with SARS-CoV-2 report increased basal levels of pro-inflammatory cytokines in pulmonary-resident aged macrophages compared to younger patients, leading to an accumulation of oxidative damage and mitochondrial dysfunction [23]. Yet, specific data on neutrophil inflammasome function in the elderly are missing. Experimentally, NLRP3 inhibition enhances overall health, mitigating multiple age-related changes associated with inflammaging. Mice with defective NLRP3 function exhibit better glycemic control, improved cognitive function, [24] decreased autophagy in neutrophils, increased phagocytosis, clearance of bacteria, and improved survival [25]. These findings have been replicated in animal models during sepsis with associated lung damage, [26] and acute hepatic damage, [27] demonstrating reduced cytokine levels, decreased neutrophilic infiltrate, and attenuated macrophage pyroptosis.

## The aged neutrophils

Neutrophils, which were traditionally considered a homogeneous population, actually consist of different phenotypes with distinct functions [28]. These various sub-populations of neutrophils likely originate from a common progenitor, but it's not yet clear whether they differentiate centrally in the bone marrow or whether they do this when in peripheral tissues. Each phenotype appears to have peculiar functions and behaves differently at different stages of the inflammatory process.

For example, one subtype of neutrophils leads to a "pro-angiogenic" response, promoting the formation of new blood vessels and aiding in the clearance of infection by generating high concentrations of matrix metalloproteinase 9, which helps remodel the extracellular matrix to facilitate tissue recovery [29]. Besides, other neutrophil subtypes appear to modulate T cells, [30] other are involved in tumor surveillance, [28] or have the ability to transmigrate from inflammatory tissues back into the bloodstream and return to the marginated pool. In homeostasis conditions, two subpopulations of neutrophils have been disclosed, although their precise functions are still unknown: cluster of differentiation (CD) 177 + neutrophils appear to be involved in anti-neutrophil cytoplasmic antibody-associated vasculitis, [31] while neutrophils expressing Olfactomedin-4, a candidate neutrophil subset marker, are associated with an increased risk of death in patients with septic shock, [32] although no substantial differences have been observed between neutrophils expressing Olfactomedin-4 and those that do not.

**Fig. 2:**
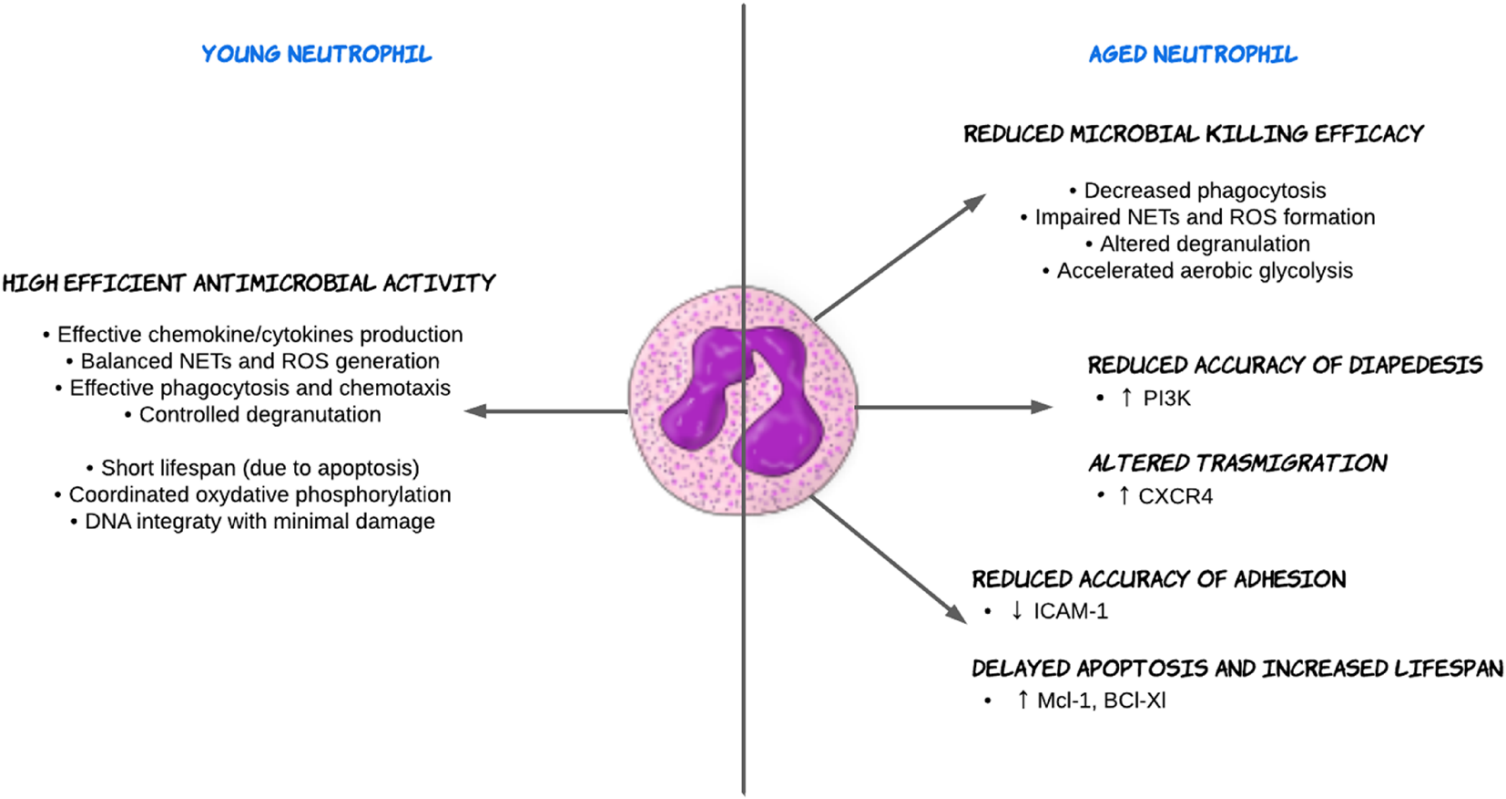
Comparison between the young and aged neutrophil. Neutrophils play a crucial role in the immune system's response to infections. In young neutrophil, there is an efficient antimicrobial activity against pathogens. In contrast, in the aged neutrophil, the immunological response might be impaired favoring several detrimental effects. The impact of aging on neutrophils can be significant and could be influenced by various factors, including overall health, genetics, and lifestyle. Understanding the changes in neutrophil function with aging is crucial for developing strategies to support immune function and mitigate age-related immuno-senescence. BCL-xl, B cell lymphoma extra large; CXCR-4, C-X-C chemokine receptor type 4; DNA, Deoxyribonucleic acid, Mcl-1, myeloid cell leukemia 1; ICAM-1, intercellular adhesion molecule 1; NET, neutrophil extracellular trap; PI3K, phosphoinositide 3-kinase ROS, reactive oxygen species

The aging of both cells and hosts is closely linked, and host aging leads to specific changes in neutrophil expression and functions. While PMNs do not divide, their senescence may lead to a process of phenotypic change with altered cytokine secretion, degranulation, cell migration, inflammation, apoptosis, and phagocytosis, making them more prone to inflammation and detrimental effects. This has been linked to organ dysfunction, diseases, and poor host outcomes. A comparison between young and aged neutrophils is illustrated in Fig. 2.

The aging of the host causes functional deficits in neutrophils that are similar to those induced by sepsis. With age, there are changes in the expression of surface markers on neutrophils, such as impaired expression of C-X-C Motif Chemokine Receptor (CXCR)-2 and CXCR4. Usually, during sepsis, neutrophils express low levels of CXCR4 to promote their redistribution toward infection sites [33]. However, in the elderly, the expression of CXCR4 increases on circulating neutrophils, directing them toward stromal-derived-factor-1-rich environments such as the bone marrow, a peculiar mechanism known as "reverse" transmigration [34].

**Fig. 3:**
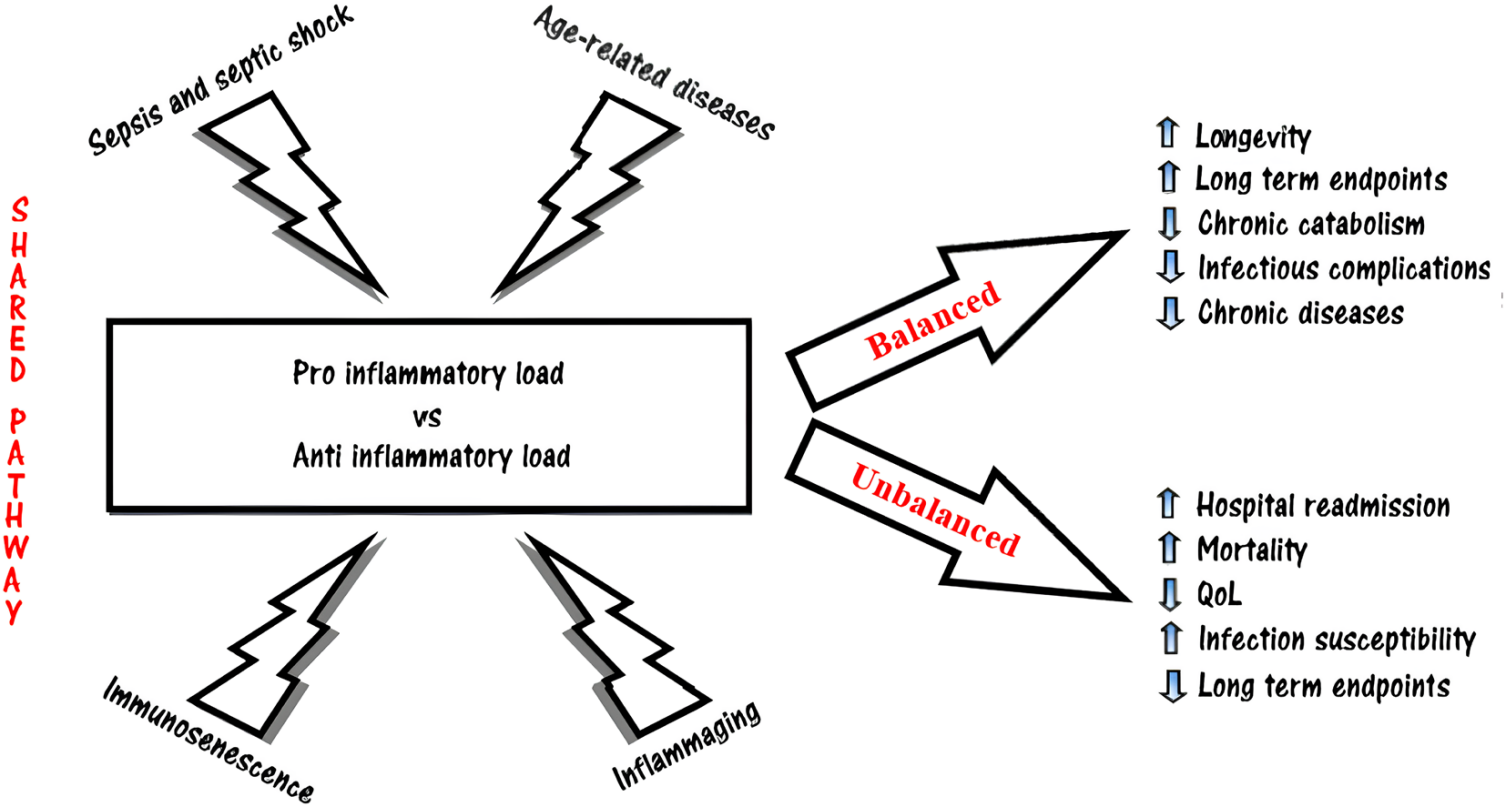
Take-home message: A shared common pathway between sepsis, aging, and inflammation. In the elderly, the chronic low-level systemic inflammation referred to as "inflammaging" is potentially linked to the development of pathological conditions such as sepsis or age-related diseases. These stimulations can disrupt the delicate equilibrium of inflammatory burden, giving rise to an elevated susceptibility to persistent, recurring, secondary, and nosocomial infections. Consequently, this heightened vulnerability contributes to amplified rates of hospital re-admissions and mortality, while concurrently diminishing the overall quality of life

Moreover, in aged mice, hematopoietic stem cells show reduced regenerative potential, and the proliferative responsiveness of immature neutrophils to granulocyte-colony-stimulating factor is altered with aging [35]. Despite this defective regenerative ability, the number of circulating neutrophils remains similar throughout life, indicating that the impairments in PMN function are mainly qualitative rather than quantitative (Fig. 3).

Indeed, accuracy of neutrophil adhesion and diapedesis mechanisms appear affected by aging. Adhesion molecules, like selectins (e.g., L-selectin) and integrins (e.g., CD11b/CD18), play a crucial role in neutrophil trafficking and recruitment to sites of inflammation. With aging, there can be variations in the expression levels and functionality of these adhesion molecules on neutrophils, which can influence their adhesion capacity and migration, potentially impacting their ability to reach infection sites.

Reduced neutrophil chemotaxis, or their ability to migrate toward inflammatory sites, has been linked to decreased expression of intercellular adhesion molecule 1 (ICAM-1). During inflammation, endothelial cells upregulate ICAM-1 expression in response to pro-inflammatory cytokines and chemokines. ICAM-1 interacts with β2 integrins (such as lymphocyte function antigen-1 and macrophage-1 antigen) expressed on neutrophils surface, facilitating their adhesion to the endothelium and subsequent transmigration into the inflamed tissue. However, in some animal studies, aging has been associated with a decline in ICAM-1 expression on endothelial cells, particularly in tissues prone to age-related pathologies. This reduction in ICAM-1 expression can impair neutrophil adhesion and subsequent transmigration across the endothelium, leading to decreased neutrophil infiltration at the site of inflammation [36].

After extravasation, following the chemo-attractive gradient, neutrophils release ROS and proteinase in order to create a path through the extracellular matrix. However, the accuracy of migration through the inflammation site is reduced in older individuals, likely due to excessive activity of phosphoinositide 3-kinases (PI3K). In response to chemotactic signals, such as chemokines or other chemoattractants, PI3K is activated and generates phosphatidylinositol (3,4,5)-trisphosphate (PIP3) from phosphatidylinositol (4,5)-bisphosphate, molecules predisposed to recruits and activates downstream effectors. An excessive PI3K activity can impact the neutrophil’s migration accuracy. Actually, a neutrophil’s overactivity is the result of elevated PIP3 levels, altering the distribution and timing of signaling events within the migrating neutrophils, leading to misdirected or prolonged migration paths [37]. Furthermore, PI3K signaling influences actin polymerization, cell polarization, and cytoskeletal rearrangements necessary for cell migration. Excessive PI3K activity can disturb these processes, resulting in impaired cell motility and reduced migration accuracy. PI3K can also interact with other signaling pathways involved in neutrophil migration, such as G protein-coupled receptors, and dysregulated PI3K activity can disrupt the balance of crosstalk between different signaling pathways, affecting the migratory response to specific chemo-attractants. The dysregulated PI3K activity and subsequent impairment of neutrophil migration accuracy observed in aging may contribute to decreased immune surveillance, delayed wound healing, and impaired resolution of inflammation [37].

Indeed, during the initial response of the immune system to pathogens, neutrophils may show reduced migration accuracy, which can impact their ability to effectively reach the site of infection. However, even when they arrive at the infection site, the resolution of inflammation can become prolonged due to several factors. One contributing factor is the defective discharge of neutrophils from the inflamed tissue, which can disrupt proper neutrophil trafficking. Neutrophils that have migrated into the site of inflammation need to be cleared or exit the tissue to prevent excessive accumulation and tissue damage. Delayed apoptosis, altered chemokine gradients, and changes in the extracellular matrix composition can all impact neutrophil egress. Indeed, altered expression of matrix metalloproteinases and tissue inhibitors of metalloproteinases can affect the degradation and turnover of extracellular matrix components, thereby impairing neutrophil egress. However, it's worth noting that the mechanisms governing these processes may not be uniform, as studies examining lung endothelia have reported contradictory findings, with ICAM-1 expression sometimes being more pronounced in the elderly, which can favor the lack of resolution of the inflammatory response [38]. During different phases of inflammation, same markers may be overexpressed while others reduced. This highlights the complexity of the aging immune system and the need to consider timing and context when studying immune modulation.

Another well-documented phenomenon in the elderly is the decreased phagocytosis of opsonized bacteria, which can be influenced by several factors altered with aging, including impaired receptor expression, such as Fc receptors and complement receptors [39]. Furthermore, dysregulated signaling pathways involving protein kinases can also impair the coordination and execution of phagocytic processes [40]. Also, increased oxidative stress and ROS production in aged neutrophils can further disrupt phagocytic machinery, leading to impaired phagocytosis and decreased intracellular killing capacity [18].

Interestingly, an increase in granule mobilization, as evidenced by the over-regulation of CD63, a primary granule marker, has been reported [37]. This suggests that certain aspects of neutrophil function may be enhanced in the elderly, while others are compromised, resulting in a complex and multifaceted effect on the immune response.

A relatively recent discovery concerns the ability of neutrophils to extrude into the extracellular environment a network of chromatin fibers and histones coated by granular proteins, such as lactoferrin, elastases, proteinase-3, and myeloperoxidase, forming structures called (NETs). The alteration of NETosis induced by aging has been poorly studied, and the results are controversial. While some studies have identified reduced NET formation by neutrophils from older donors, the current understanding suggests an increase in NET production in elderly subjects [41]. However, although the overall production of NETs is higher in elderly individuals, functionality and effectiveness of these NETs may be compromised due to alterations in their composition and impaired clearance mechanisms.

In aging, neutrophils' production of ROS becomes dysregulated, even in the absence of infection or stimuli. This increased ROS production can trigger spontaneous NET release, contributing to the elevated basal levels of NET formation in aged individuals [42]. Such a phenomenon is probably related to the chronic low-grade inflammation and altered neutrophil activation observed in older individuals. Consequently, the increased release of NETs in the absence of infection or appropriate stimuli can lead to prolonged inflammation and tissue damage, forming a vicious cycle. Furthermore, NETs released by aged neutrophils generally have an unbalanced composition of antimicrobial proteins, including altered levels of DNA and histones [43]. For example, increased citrullination of histones, mediated by peptidyl-arginine deiminase enzymes, has been reported in aged neutrophils. These modifications can affect the ability of histones to bind DNA and promote chromatin de-condensation, potentially influencing NET structure and function.

While NETs are designed to trap and eliminate pathogens, dysregulated NET formation in aging may compromise their antimicrobial function. Altered composition, reduced antimicrobial capacity, and impaired clearance can undermine the ability of NETs to effectively control infections, leading to increased susceptibility to microbial pathogens. Moreover, in elderly patients exposed to infection, NETs may exhibit an enhanced pro-inflammatory phenotype. This can be attributed to alterations in the composition of NETs, including increased exposure of pro-inflammatory molecules such as high-mobility group box 1 protein [13, 44]. These modified NETs can activate immune cells, including macrophages and dendritic cells, leading to sustained inflammation and tissue damage. As such, the increased basal levels of NET formation in aging individuals can have both beneficial and detrimental effects. On one hand, NETs can help in the clearance of pathogens and contribute to immune defense. On the other hand, the elevated levels of NETs in the absence of specific stimuli can lead to prolonged inflammation, tissue damage, and potentially contribute to age-related pathologies.

## How sepsis impacts on the innate immune system: a focus on neutrophil function

During the course of sepsis, several alterations in the immune system have been reported, affecting both innate and adaptive responses [8]. Sepsis-related immune suppression is marked by significant impairments that share similarities with immuno-senescence, as shown in Table 1. As mentioned earlier, sepsis exerts its impact on the immune system by disrupting the functionality and quality of effector cells responsible for maintaining homeostasis [11].

In sepsis, an initial over-expression of pro-inflammatory cytokines by various components of the innate immune system was reported. This exaggerated and uncontrolled activation of the inflammatory response can promote the activation of the coagulation system and the formation of micro-thrombi, potentially causing dangerous conditions such as disseminated intravascular coagulation [45]. The reciprocal interaction between immune system cells and platelets, endothelial cells, and clotting factors is referred to as "immunothrombosis" with roles in preventing pathogen spread. During sepsis, immune cells interact with endothelial cells, inducing a pro-adhesive and pro-thrombotic phenotype rich of tissue factor (TF), E-selectin, ICAM-1, vascular cell adhesion molecule 1 (VCAM-1), and von Willebrand factor (vWF). In conditions featuring endothelial dysfunction, such as cardiovascular disease and sepsis, the induction of NETosis further fuels immuno-thrombosis mechanisms. Specifically NETs, harbor IL-1α and other DAMPs on their chromatin web, causing upregulation of vWF and TF expression by the endothelium, increasing thrombin production from platelets, and blunting the production of endogenous anti-coagulant factors [46, 47].

Examining various neutrophilic phenotypes has revealed that the presence of aged neutrophils in the bloodstream is harmful to tissues and promotes vascular inflammation. In an ischemia–reperfusion model study, the depletion of aged neutrophils prevented micro-clot formation and improved survival after myocardial infarction [48]. While NETs may contribute to thrombosis, this study did not identify them as the primary instigator, as two different NET inhibitors failed to prevent clot formation in re-perfused microcirculation. Furthermore, no evidence was found to suggest that the proliferation and apoptosis of endothelial cells are directly altered by neutrophils, indicating that aged neutrophils do not compromise basal vascular integrity before a direct insult [48].

However, the severity of organ dysfunction and the increased mortality cannot be solely explained by the pro-inflammatory status alone. Simultaneously, sepsis induces an increased release of anti-inflammatory cytokines that alter the ability of innate immune system cells to respond to infections, leading to a reduction in several essential functions. For example, during sepsis, there is a decreased expression of the HLA-DR isotype in the bone marrow, which impairs the antigen-presenting capabilities of monocytes, further exacerbating immunosuppression and reducing the ability to combat pathogens [49].

Other mechanisms of immune system dysregulation during sepsis include the reduction of antimicrobial activities of macrophages and neutrophils. These alterations result in impaired recruitment and migration to the site of infection, as well as impaired ability to phagocytose foreign materials. In addition, there is also an increased loss of effector immune cells, wherein the process of apoptosis is affected, increased in macrophages and dendritic cells, and delayed in PMNs. Generally, apoptosis aims to eliminate damaged cells in order to maintain homeostasis but, in sepsis, the death receptor ligands increase the expression of Fas/Fas-ligand on the surface of the peripheral blood mononuclear cells leading to downstream activation of Caspase-8 and ultimately Caspase-3, also called as the extrinsic apoptosis pathway [50]. As a result, the immune system faces greater challenges in fighting pathogens, leaving the patient more vulnerable to secondary infections. In elderly individuals, these mechanisms of immunosuppression, resulting from both sepsis and inflammaging, overlap and act synergistically, likely remaining among the main causes of poor outcomes in septic patients.

After the migration toward the site of infection, neutrophils employ various mechanisms to protect the body. Some of these mechanisms, like phagocytosis, oxidative burst, cytokine release, and degranulation, have long been known, while others, such as neutrophil extracellular trap formation and microvesicle release, have been more recently characterized. While in the early stages of infection, especially in non-severe disorders, the antimicrobial response of neutrophils is adequate and effective, in the more severe forms such as septic shock, under a condition of prolonged stimulation and immune suppression, the same mechanism can become impaired. In fact, during severe infection, neutrophils show defective recruitment and migration to the infection site, which can be attributed to a higher proportion of immature cells in the bloodstream [51]. This condition is influenced by the activation of pro-inflammatory cytokines and toll-like receptors, leading to the desensitization of chemokine C-X-C motif ligand 12, a key regulator of neutrophil mobilization, and promoting their release into the bloodstream. Furthermore, the receptor CXCR2 appears to be involved as an antagonist of CXCR4, further promoting neutrophil mobilization [33].

Moreover, in the course of sepsis, C–C chemokine receptor type (CCR) 2 signaling is implicated in recruiting neutrophils to infected tissues. The release of CCR2 ligands, particularly monocyte chemoattractant protein-1, from infected tissues promotes the activation and migration of neutrophils through a CCR2-dependent mechanism. While this is crucial for host defense against pathogens, dysregulated CCR2 signaling can lead to excessive and inappropriate neutrophil infiltration, causing tissue damage [52].

Furthermore, the chemokine gradient is vital for neutrophil diapedesis through endothelial tight junctions. However, during severe sepsis, neutrophils exhibit reduced affinity for chemoattractants, possibly related to pro-inflammatory products that, together with chemokines released from the luminal part of the endothelium, induce conformational changes on the surface of the neutrophils [53].

While some populations of the innate immune system, such as natural killer cells, dendritic cells, and macrophages, increase their apoptosis rate during sepsis, neutrophils demonstrate delayed apoptosis, attributed to the increased amount of anti-apoptotic proteins, particularly myeloid cell leukemia 1 (Mcl-1) [54]. Pro-inflammatory mediators like lipopolysaccharide activate anti-apoptotic pathways, resulting in higher expression of anti-apoptotic proteins, such as B cell lymphoma-extra-large and Mcl-1 proteins [55]. This might explain the increased neutrophils’ lifespan during sepsis [56]. Moreover, the antimicrobial activities of the innate system are severely impaired during sepsis. This is characterized by reduced production of pro-inflammatory cytokines and a tendency for the reduction of anti-inflammatory mediators. In certain cases, microorganism-specific stimuli (e.g., during *Pseudomonas aeruginosa* sepsis) may activate the complement system, increasing component 5a while decreasing ROS and nitric oxide production, as well as phagocytosis [56].

Relatively recent studies have indicated dysregulated NET formation following severe infection [57]. While NETs play a crucial role in defense mechanisms, their dysregulation during sepsis can have both positive and negative consequences. Although they prevent bacterial dissemination in the early stage of infection, promoting the physical containment of microbes, their presence during sepsis is associated with more severe disease and organ damage [58]. Additionally, NETs may contribute to coagulation disorders in sepsis, particularly in the liver sinusoids and pulmonary capillaries, as their components interact with activated platelets, promoting adhesion, activation, and aggregation, leading to thrombosis, cellular ischemia, and organ injury [59].

Lastly, degranulation is qualitatively impaired during sepsis, resulting from an alteration in granule contents and balance in neutrophils. This leads to heterogeneity in granules and subsequent impairment of related neutrophil functions, such as migration and diapedesis [60].

## Time is the key element for immunomodulation of neutrophils during sepsis

Sepsis and septic shock are serious public health concerns. To address this issue, implementing a diagnostic process for sepsis is necessary to identify patients at higher risk of death, optimizing therapeutic strategies to reduce mortality and associated costs. Nevertheless, a large number of randomized trials were organized in the hypothesis of modulating the septic response to infection, but none of these demonstrated to increase long-term survival.

Nowadays, a significant portion of sepsis-related mortality occurs several days or months after a patient's discharge, probably due to the immunosuppression state typical of septic patients, coupled with their comorbidities. This phenomenon is particularly pronounced in the elderly population, possibly due to immuno-senescence. As a result, ongoing trials are focusing on immune-modulatory therapies. Understanding how sepsis affects the functions of the innate immune system could be the key to identifying appropriate therapeutic strategies that can prevent long-term mortality. Neutrophils play a crucial role in the fight against pathogens, exhibiting different activities depending on the phase of the septic process [61]. Targeting these cells may help restore a balanced immune response to the infection.

Immuno-modulating agents are commonly used in clinical practice and have significantly improved the prognosis of several diseases, such as inflammatory bowel diseases, rheumatoid arthritis, psoriasis, chronic obstructive pulmonary disease, and anti-neutrophil cytoplasmic antibody-associated vasculitis. Several trials are currently focusing on targeting neutrophils as a therapeutic strategy for different diseases, but little emphasis has been given to their modulation in sepsis. Some attempts have been made to use immunosuppressant treatments for neutrophils or their environment in septic patients, but uncertainty surrounds their effectiveness in recent years [62]. Many trials failed to show the benefits of using such treatments in clinical practice. For example, trials concerning the granulocyte–macrophage colony-stimulating factor, which augments circulating neutrophil levels, did not prove useful despite promising preclinical results [63]. Similarly, activated protein C, which can reduce integrin-mediated migration and NETs formation in neutrophils in preclinical settings, in recent trials did not show the clinical benefits of immunomodulation in septic patients [64].

Similarly, anti-TNF-α drugs, which are effective in treating chronic diseases like rheumatoid arthritis, have been considered as potential targets in animal models of sepsis [65]. However, skepticism arose due to their seemingly irrelevant or even harmful effects in the clinical setting. Though, immuno-modulating agents might require several days before determining substantial effects, especially if a large quantity of cytokines is produced abruptly as it happens in sepsis [62].

In fact, one of the main differences between experiments in animal models and clinical trials is the time of anti-TNF-α administration. In most of the pre-clinical studies, anti-TNF-α drugs are administered around the onset of sepsis [62]. Conversely, the belated administration of these drugs is ineffective even in animal models [66]. Thus, an earlier administration of anti-TNF-α might be investigated in future clinical studies even though finding the right time of administration could be challenging. A relatively recent meta-analysis showed that the use of anti-TNF-α antibodies reduces 28-day mortality compared with controls in septic patients [67]. Thus, the use of anti-TNF-α might require further evaluation, especially concerning the right time of administration with adequate sample size trials. Stratification of patients based on early markers of sustained inflammatory response might further help in findings people who would benefit the most from immunomodulatory approaches.

Actually, the early hours of sepsis are crucial for initiating an appropriate treatment even though only modest symptoms might appear in the initial phases. Especially in the elderly, only an obnubilated mental status can be the very first signal of sepsis [68]. Typical symptoms tend to manifest later in the course of the disease in these frail patients, with the risk of milder response to the administered treatments. In the elderly, the rationale might be that of increasing neutrophils’ activity during the very early phases for favoring the immune response toward pathogens, and that of reducing neutrophils over-activity during the late phases to prevent detrimental effects.

## Restoring neutrophils activity

Table 2 presents recent preclinical evidence regarding new potential neutrophil-related targets or treatments to restore their activity. One example is IL-33, a cytokine belonging to the IL-1 family, mainly known for its role in T_H_2 polarization. Sepsis is associated with a reduction of the IL-33 receptor during the early phases, leading to a decreased number of neutrophils at the site of infection [69]. The administration of IL-33 or other molecules that counterbalance its activity promotes higher neutrophil infiltration at the infection site and results in improved prognosis in animal models of sepsis, as reported in Table 2.

**Table 2:** Recent pre-clinical evidence of treatments/targets for restoring neutrophils activity

Treatment/Target	Author	Comment
ADAM 17	Mishra et al.[84]	Mice lacking metalloprotease-17 have a higher infiltration of neutrophils into the site of infection and reduced pro-inflammatory cytokines and bacterial in the bloodstream
BTLA	Kobayashi et al.[85]	Anti-BTLA antibodies treatment decreases the risk of endotoxic shock and increases the survival
CXCL1 and CXCL2	Craciun et al.[86]	Intra-peritoneal administration of CXCL1 and CXCL2 favorites an early infiltration of neutrophils into the site of infection, less risk of peritoneal bacteria, and a better outcome
CD 44	Hasan et al.[87]	Administration of antibodies directed toward CD44 results in an augmented inflow of neutrophils into the site of infection
IL-5	Xu et al.[88]	Administration of IL-5 in IL-33-deficient mice induces a higher recruitment of neutrophils into the site of infection
IL-7	Kasten et al.[89]	Administration of IL-7 augments the neutrophils enrollment and killing of pathogens
IL-15	Pelletier et al.[90]	Administration of IL-15 reduces neutrophils apoptosis
IL-33	Alves-Filho et al.[91] Lan et al.[92]	Administration of IL-33 favors the infiltration of neutrophils in the site of infection via up-regulation of CXCR2
IL-34	Lin et al.[93]	Administration of IL-34 favors the infiltration of neutrophils in the site of infection
Mesenchymal stromal cells	Hall et al.[94]	Administration of mesenchymal stromal cells favorite neutrophils phagocytosis and reduces multi-organ failure
PI3K	Sapey et al.[37]	Inhibition of PI3K favors a normal migration of neutrophils
Trimetazin	Chen et al.[95]	Administration of trimetazin favorites neutrophils arrival into the heart tissue because of CXCR2 up-regulation

*ADAM 17* A disintegrin and metalloprotease 17, *BTLA* B- and T-lymphocyte attenuator, *CXCL 1 and 2* chemokine (C-X-C motif) ligand 1 and 2, *CXCR2* C-X-C Motif Chemokine Receptor 2, *IL-5* interleukin 5, *PI3K* phosphoinositide 3-kinase

Conversely, immunosuppression contributes to increased mortality in the later stages of sepsis. Detecting the up and down regulation of surface molecules on PMNs’ can aid in the stratification of patients’ risk of mortality. Among these molecules, CD24 and CD279 appear to be optimal biomarkers for predicting subsequent sepsis and worse outcomes. CD24 is a small, heavily glycosylated cellular surface protein predominantly expressed most significantly in neutrophils, connected to the membrane. CD279, also known as programmed death 1 (PD-1), is a protein found on the surface of B and T lymphocytes that regulate T cell responses, acting as a suppressor of neutrophil stimulation. In sepsis, the cross-linking of CD24 on the neutrophil surface is downregulated, resulting in delayed apoptosis [70]. Conversely, higher expressions of CD24 and CD279 on neutrophils predict clinical worsening of sepsis, whereas lower expressions are associated with earlier discharge from the hospital [71]. Similar to the promising results seen in cancer treatment with immune checkpoint targeting, targeting PD-1 in sepsis seems promising based on recent evidence [72, 73]. Also in progressive multifocal leukoencephalopathy, PD-1 blockade with pembrolizumab could reduce viral load and increase lymphocyte activities [74]. An observational study demonstrated that anti-PD-1 and programmed death-ligand 1 (PD-L1) antibodies enhance neutrophil functionality [75].

Lastly, in older adults, the accuracy of neutrophil migration through the inflammatory site may be reduced, possibly due to excessive PI3K activity. Blocking PI3K, particularly the inhibition of PI3Kγ or PI3Kδ, has been linked to the restoration of cell migration ability [37].

## Reducing neutrophils detrimental effects

As the fight against pathogens reiterates, the risk of tissue and organ damage increases. In general, neutrophils’ activity can determine several multi-organ failure in different ways: releasement of proteolytic enzymes, NETs, and blood flow occlusion because of excess neutrophils’ binding to the endothelium. As shown in Table 3*,* recent evidence demonstrates that animal models of sepsis with reduced production of NETs have a better prognosis, fewer organ damages, and improved survival.

**Table 3:** Recent, preclinical evidence of treatments/targets for reducing neutrophils over-activity

Treatment/Target	Author	Comment
Annexin	Vago et al. [96]	Administration of annexin A1-active N-terminal peptide induces neutrophils apoptosis
CCR2	Souto et al. [52]	Mice with reduced CCR2 activity, either genetically or via treatments, showed less organ damages and mortality
CD-40L	Rahman et al. [97]	Mice deficient of CD-40L showed less neutrophil migration into the septic lungs and MPO activity
CDK	Leitch et al. [98]	Administration of R-roscovitine augments neutrophil apoptosis via inhibition of CDK7 and CDK9
CDK9	Wang et al. [99]	Administration of flavopiridol inhibits CDK9, favoring neutrophils apoptosis
CIRP	Ode et al. [100]	CIRP^−/−^ mice have a higher survival trend
Doxorubicin	Zhang et al. [101]	Administration of selective doxorubicin-conjugated nanoparticles induces neutrophils apoptosis, favoring a better outcome
Inflammasome	Bakele et al. [102]	Targeting neutrophils inflammasome can reduce inflammation during sepsis since it is related to the production of IL-1β
IL-3	Weber et al. [103]	Mice lacking IL-3 have less risk of sepsis
LFA-1 and Mac-1	Asaduzzaman et al. [104] Herter et al. [105]	Administration of antibodies against LFA-1 and Mac-1 decreases neutrophil infiltration of septic lung tissues and prevents AKI
LOX-1	Wu et al. [106]	Deletion of LOX-1 reduces neutrophils overactivity but improves bacterial clearing
JAM-C	Hirano et al. [107]	Administration of JAM-C antibodies prevents acute lung injury but reduces neutrophil apoptosis in septic mice
NETs	Mai et al. [108]	Administration of DNase after 4 h from sepsis induction reduces cell-free DNA, IL-6 levels, but augments IL-10, and prevents organ damage
	Martinod et al. [109] Biron et al. [110]	Mice deficient of PAD4, and therefore deficient of NETs production, show a reduction of organ damages, are protected from endotoxemia shock, and have an increased survival
	Li et al. [111]	Inhibition of citrullinated histone H3 increases the survival of mice with sepsis
NLRP3	Luo et al. [26]	Hemin inhibits NLRP3 inflammasome activation, resulting in a reduction of neutrophil infiltration and mitigating sepsis-induced acute lung injury
	Wu et al. [27]	Silencing of the NLRP3 gene decreases hepatic cytokine levels, mitigates neutrophil infiltration, and prevents macrophage pyroptosis, leading to attenuation of hyper-bileacidaemia induced by sepsis
P-selectin, E-selectin	Herter et al. [105]	Administration of antibodies against P-selectin or E-selectin prevents AKI in animal models of sepsis
Resolvin	Kebir et al. [112]	Resolvin administration reduces lung injuries in animal models of sepsis favoring the elimination of activated neutrophils
TICAM2	Lin et al. [113]	Mice lacking TICAM2 showed less organ damages during systemic inflammation
Ticagrelor	Rahman et al. [114]	Ticagrelor prevents neutrophils from infiltrating lung tissues during sepsis
Tyrosine kinase inhibitor (dasatinib)	Futosi et al. [115]	Mice treated with dasatinib showed reduced neutrophil migration into the site of infection but not alter their anti-bacterial activity
	Gonçalves-de-Albuquerque et al. [116]	Administration of dasatinib favors a better outcome and reduces risks of organ damages in mice with sepsis

*AKI* acute kidney injury, *CCR2* C–C chemokine receptor type 2, *CD* cluster of differentiation, *CD-40L* cluster of differentiation 40 ligand, *CDK* cyclin-dependent kinases, *CIRP* cold-inducible RNA-binding protein, *IL* interleukin, *JAM-C* junctional adhesion molecule-C, *LFA-1* lymphocyte function antigen-1, *LOX-1* lectin-like oxidized low-density lipoprotein receptor 1, *Mac-1* macrophage-1 antigen, *MPO* myeloperoxidase, *NET* neutrophil extracellular trap, *NLRP3* NOD-, LRR- and pyrin domain-containing protein 3, *PAD4* peptidyl arginine deiminase 4, *TICAM2* toll-like receptor adaptor molecule 2

The increase of neutrophil apoptosis is another potential therapeutic approach. Normally, neutrophils initiate the apoptotic process after eliminating pathogens, leading to the resolution of inflammation [76]. However, apoptosis of neutrophils tends to be decreased in septic patients [77]. Possible explanations include hyper-activation of nuclear factor-kappa B and downregulation of caspases-9 and -3 in neutrophils of septic patients, as well as the accumulation of cytoplasmic myeloid nuclear differentiation antigen, which slows the apoptotic process in these neutrophils [78]. Treatment with molecules that prevent apoptosis, such as caspase inhibitors or anti-apoptotic cytokines, has been shown to improve survival in sepsis, especially in monocytes [78].

Another potential target process is the neutrophil recruitment and infiltration into the site of infection. CCR2 signaling on neutrophils triggers a cascade of intracellular events that facilitate their migration. Upon ligand binding, CCR2 activates downstream signaling pathways, such as the mitogen-activated protein kinase pathway PI3K pathway, leading to actin polymerization and cytoskeletal rearrangement. These events promote neutrophil chemotaxis and adhesion to endothelial cells, facilitating their transmigration into the infected tissues. Preclinical studies showed that blockade of CCR2, either genetically or pharmacologically, has the potential to reduce the inappropriate infiltration of neutrophils during sepsis, leading to organ damage and mortality [52].

Recent studies show that propofol, one of the most commonly used anesthetic drugs, can prevent neutrophil overactivity by inhibiting formyl peptide receptor 1 [79]. In fact, the formyl peptide receptor 1 is related to neutrophils chemotaxis, superoxide production, elastase, and leukotriene B4 release [79]. However, not all studies have found propofol to be an anti-neutrophil drug, as low-dose treatments have been shown to have a pro-survival effect on neutrophils. [80].

Another therapeutic strategy involves removing leukocytes or pro-inflammatory molecules from the blood of septic patients. A prospective, multicenter study demonstrated that patients treated with hemodiafiltration had longer survival rates compared to their predicted survival based on the APACHE II (Acute Physiology and Chronic Health Evaluation II) score [81]. On the contrary, leukofiltration showed no difference in terms of mortality but improved renal function in septic patients [82]. However, generalizations are not possible at the present moment due to limited data availability, as reported in a recent meta-analysis [83]. These therapeutic strategies hold promise in addressing the complexities of sepsis and its impact on the immune system, but further research is needed to establish their effectiveness and safety in clinical settings.

In conclusion, a novel therapeutic approach could target inflammasomes, acting upstream of the production of specific cytokines. Specifically, inhibiting the activation of inflammasome NLRP3 in response to the accumulation of DAMPs holds promise for controlling systemic chronic inflammation in the elderly and addressing age-related pathologies, extending beyond the neutrophilic level.

Pre-clinical studies provide evidence that the administration of Hemin, [26] an inhibitor of heme oxygenase-1 (HO-1), effectively hinders the activation of inflammasome NLRP3. This intervention results in reduced levels of IL-1β and IL-18, accompanied by the inhibition of neutrophil recruitment. Notably, this limits the inflammatory response and mitigates acute lung damage induced by sepsis. Additionally, exploring the potential of gene silencing of NLRP3 [27] reveals promising outcomes, as it appears to diminish neutrophilic infiltrate and associated liver damage induced by sepsis. These findings suggest the potential of targeting inflammasome NLRP3 as a strategy to address age-related pathologies and sepsis-induced complications.

## Concluding remarks and future perspectives

Functional neutrophils are of pivotal importance for host defense during infective conditions. Yet, sepsis itself may cause neutrophil dysfunction reducing the appropriate release of pro-inflammatory mediators in the early phase in favor of a more prolonged action with deleterious side-effects. Such an effect is particularly seen in the elderly as it couples with the general aging of the inflammatory system, which has similar effects on PMN function.

A more profound understanding of how the innate immune system becomes impaired during sepsis and aging is essential in order to improve patients’ health. In the near future, advancements in understanding neutrophil functions are eagerly awaited to develop potential immunomodulatory therapies that can enhance clinical outcomes. Even though this concept may be counterintuitive, we here reported how sustained inflammation and neutrophil activation especially in the late phase of an infection can be deleterious. However, the short life of these cells together with the presence of different phenotypes (including regulatory ones) does not encourage approaches targeting those cells at broad. Most probably, a personalized therapy taking into account the age of the patients as well as a tight timing control targeting specific mediators/mechanisms (i.e., NETosis or inflammasomes) may be the avenue to follow.

## Data Availability

This is a review article. It does not include any original data.
